# A large-scale fMRI dataset for human action recognition

**DOI:** 10.1038/s41597-023-02325-6

**Published:** 2023-06-27

**Authors:** Ming Zhou, Zhengxin Gong, Yuxuan Dai, Yushan Wen, Youyi Liu, Zonglei Zhen

**Affiliations:** 1 State Key Laboratory of Cognitive Neuroscience and Learning & IDG/McGovern Institute for Brain Research, Beijing Normal University, Beijing, 100875 China; 2 Beijing Key Laboratory of Applied Experimental Psychology, Faculty of Psychology, Beijing Normal University, Beijing, 100875 China

**Keywords:** Perception, Extrastriate cortex

## Abstract

Human action recognition is a critical capability for our survival, allowing us to interact easily with the environment and others in everyday life. Although the neural basis of action recognition has been widely studied using a few action categories from simple contexts as stimuli, how the human brain recognizes diverse human actions in real-world environments still needs to be explored. Here, we present the Human Action Dataset (HAD), a large-scale functional magnetic resonance imaging (fMRI) dataset for human action recognition. HAD contains fMRI responses to 21,600 video clips from 30 participants. The video clips encompass 180 human action categories and offer a comprehensive coverage of complex activities in daily life. We demonstrate that the data are reliable within and across participants and, notably, capture rich representation information of the observed human actions. This extensive dataset, with its vast number of action categories and exemplars, has the potential to deepen our understanding of human action recognition in natural environments.

## Background & Summary

Human action recognition is one of our critical capacities. The capacity enables us to effortlessly identify various actions performed by others within a single glance and thus easily fulfill the human-environment and human-human interactions in daily life. Over the past several decades, significant strides have been made in understanding the neural mechanisms of human action recognition^1–12^. Many brain areas have been identified as playing a role in processing information from observed actions, including the ventral visual pathway that processes object and body identity and category^12–14^, the lateral visual pathway that processes dynamics of object appearance and conceptual information^14,15^, and the dorsal visual pathway that processes spatial relationships between objects and human body to guide action visually^16,17^. However, most neuroimaging studies on action recognition use well-controlled images and videos with few action categories in simple contexts^6–12^. As neural responses to stimulus are primarily modulated by the contexts^18–20^, it is unclear whether the findings from the controlled actions can be well generalized to diverse actions from real-life scenarios.

Large-scale neuroimaging data with naturalistic stimuli have been collected to improve our understanding of how the brain perceives the dynamic and interactive world^21–25^. These datasets often use continuous movies as stimuli, which contain rich human activity and thus can be used to examine the functional organization of the brain for social interaction in everyday life^26–30^. However, lacking proper annotations of human actions for these movie stimuli limits the application of these data in testing specific hypotheses related to action recognition. To our knowledge, only two large-scale neuroimaging datasets have been specifically designed for understanding the neural basis of human action recognition under naturalistic contexts. Dima and her colleagues find that visual, action, and social-affective features predict neural patterns at early, intermediate, and late stages, respectively, curating large-scale sets of naturalistic videos of 18 everyday actions and electroencephalography recording^4^. Tarhan and Konkle measure brain responses to 60 everyday actions with functional magnetic resonance imaging (fMRI) and reveal that the human action representations are primarily driven by sociality and interaction envelope^5^. Although both data are publicly available, large-scale functional magnetic resonance imaging (fMRI) datasets for human action recognition, in which the stimuli are sampled from various real-world contexts and richly annotated, are still urgently needed.

To address this challenge, we present Human Action Dataset (HAD), a large-scale fMRI dataset recorded from 30 participants while viewing 21,600 video clips. The clips were selected from the Human Action Clips and Segments (HACS) dataset, a comprehensive video benchmark for human activity understanding created by the field of computer vision^31^. HACS Clips are sampled from 504 K videos retrieved from YouTube, encompassing a wide range of complex human activities in daily living. Each clip lasts two seconds and is annotated according to a taxonomy of action categories. We demonstrated that recorded fMRI responses for the observed human actions show high within-subject reliability and between-subject consistency. Moreover, we revealed that the data capture rich representation information of the observed human actions. With its extensive collection of action categories and exemplars, we believe that HAD has the potential to advance our understanding of visual action representation in natural settings.

## Methods

### Participants

Thirty students (mean ± standard deviation [SD] of age: 22.17 ± 2.25 years, 17 females) from Beijing Normal University took part in the HAD experiment (sub01-sub30). The participants had normal or correct-to-normal visual acuity. All participants provided informed written consent for their participation and sharing their anonymized data. The study was approved by the Institutional Review Board of Beijing Normal University (approval number: ICBIR_A_0111_001_02).

### Stimuli

**Fig. 1:**
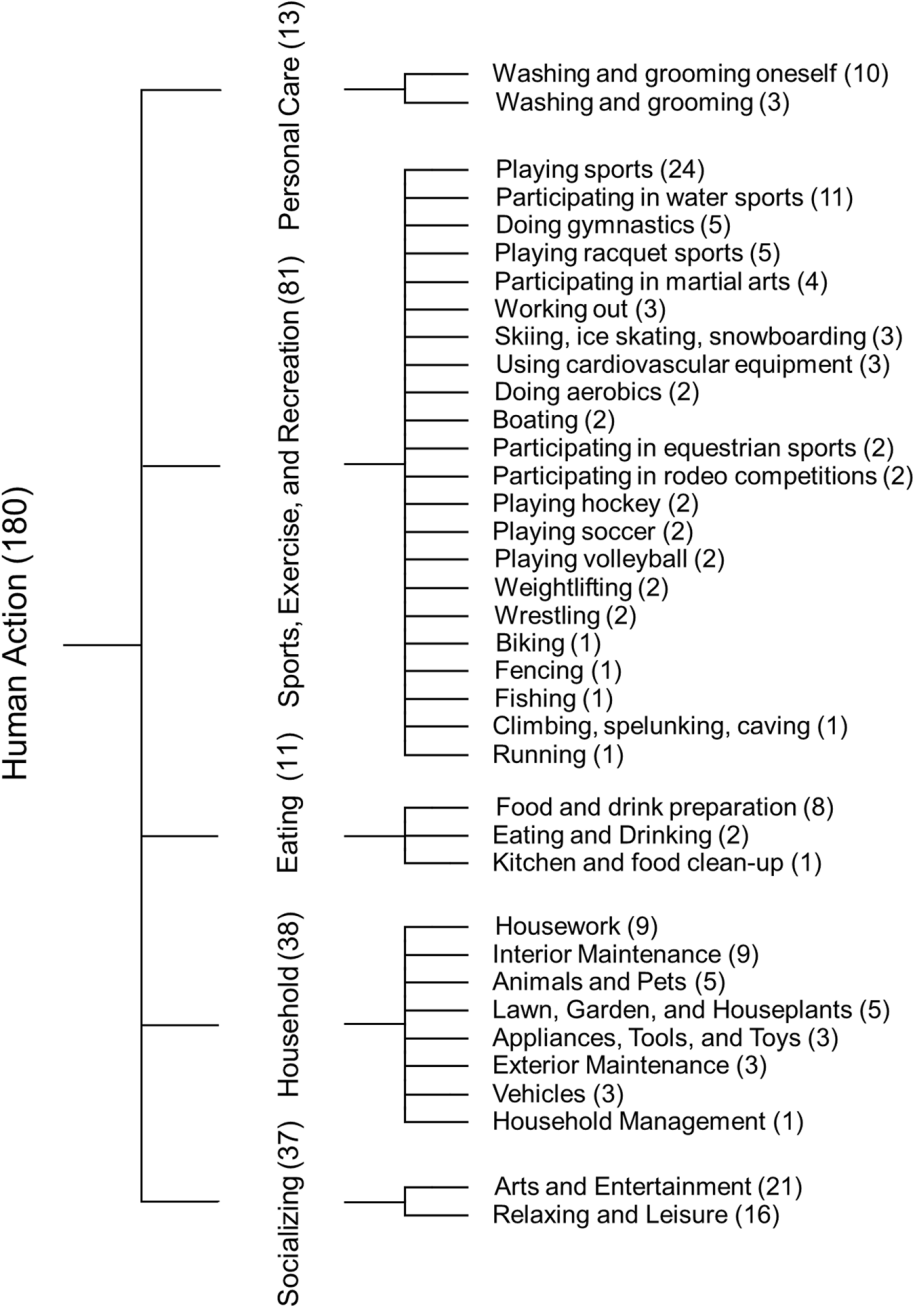
The hierarchy structure of action categories for Human Action Dataset (HAD). The 180 action classes are organized in a hierarchy tree with four levels of depth. The first three levels are shown here and leaf information can be found in Supplementary Table 1. The figures in parentheses indicate the number of its subordinate categories.

The stimuli of human actions were selected from Human Action Clips and Segments (HACS) dataset. HACS is a large-scale video dataset designed as a benchmark for evaluating the performance of state-of-the-art computer vision models in human action recognition and temporal localization^31^. HACS utilizes a taxonomy of 200 action classes, covering a wide range of complex human activities in daily life^32^. HACS consists of two kinds of manual annotations: HACS Clips and HACS Segments. HACS Clips contains 1.55 M two-second clip annotations sampled from 504 K untrimmed videos; HACS Segments contains 139 K action segments densely annotated in 50 K untrimmed videos, where both the temporal boundaries and the action labels of action segments are annotated. Although both types of annotation share the same taxonomy of 200 action classes, they are designed for different purposes. HACS Clips is designed for action recognition whereas HACS Segments is designed for temporal action localization. Because our aim is to collect fMRI data for human action recognition, we chose HACS Clips as the stimuli for the HAD experiment. HACS Clips includes both positive and negative examples. That is, each clip has been annotated to indicate whether a target action really happens (i.e., positive) or not (i.e., negative). As the positive clips are the desired stimuli for our fMRI experiment, twenty of the 200 action categories were excluded due to having too few positive examples ( < 480). The remaining 180 action categories were structured around a semantic ontology defined by ActivityNet^32^, which organizes activities according to social interactions and where they usually take place (Fig. 1). For these 180 categories, we implemented a four-pronged procedure to select representative and high-quality clips from the large pool of HACS Clips. First, the clips with disproportionate aspect ratios (three SD away from the mean value) were excluded from the HACS Clips pool. Second, 120 positive video clips were randomly selected from the pool for each category. Third, ten human raters were recruited to visually inspect and mark if a target action was easy to recognize from each clip. Each rater was assigned to check 18 categories of human actions (120 samples/category) which were not overlapping among raters. On average, five clips were detected as hardly identifiable across the 180 categories of actions. However, it was revealed that some action categories show much more unrecognized samples than others (Supplementary Fig. S1), indicating that visual inspection is very necessary to select qualified stimuli for the subsequent fMRI experiment. Finally, the clips from which the target action was hard to be recognized were replaced by a qualified positive clip randomly selected from the pool of HACS positive clips. As a result, 21,600 HACS clips were selected as our stimuli, with 120 unique clips for each of the 180 action categories.

### Experimental design

Each of the 30 participants completed a rapid event-related fMRI experiment for human action recognition. The experiment consisted of 12 runs, and 60 distinct video clips (one clip/category) were presented in each run. The 180 categories cycled every three runs, and each action category was thus repeated four times in a session. The stimuli sequence of 180 clips (categories) was optimized using Optseq (https://surfer.nmr.mgh.harvard.edu/optseq/) to prevent consecutive appearances of clips from the same superordinate category and evenly divided into three runs. A clip was presented 2 seconds followed by a 2-second interval and a blank trial was inserted after every five trials, with four blank trials added at the beginning and end of each run. Consequently, each run lasted 5 minutes and 12 seconds. The clips were completely distinct for each run and participant in order to sample brain response to video clips as much as possible. That is, each participant viewed 720 unique human action videos, and 21,600 videos were viewed in total across 30 participants. All stimuli were presented using Psychophysics Toolbox Version 3 (PTB-3)^33^ via an MR-compatible LCD display mounted at the head end of the scanner bore. The videos were presented at the 16° × 16° visual angle. Participants viewed the display through a mirror attached to the head coil. Participants were asked to fixate on the dot in the center of the screen and press one of two response buttons as quickly as possible after a clip disappeared to indicate that the human action presented in the clip was a sport or a non-sport action. Specifically, they were instructed to press a button with their right thumb for a sport action and press another button with their left thumb for a non-sport action.

### MRI acquisition

MRI data were acquired on a Siemens MAGNETOM Prisma 3 Tesla (3 T) MRI scanner at the BNU Imaging Center for Brain Research (Beijing, China) equipped with a 64-channel phased-array head coil. Task fMRI, field map, and structural MRI were acquired in a scan session lasting approximately 1.5 hours. Earplugs were used to attenuate scanner noise, and extendable padded head clamps were used to restrain head motion. No physiological data (e.g., heartbeat and breathing rates) were recorded.

#### Functional MRI

Bold-oxygenation-level-dependent (BOLD) fMRI data were collected using a Siemens multi-band, gradient-echo accelerated echo-planar imaging (EPI) T2*-weighted sequence: 72 slices co-planar with the AC/PC; in-plane resolution = 2 × 2 mm; 2 mm slice thickness; field of view = 200 × 200 mm; TR = 2000 ms; TE = 34 ms; flip angle = 90°; bandwidth = 2380 Hz/Px; echo spacing = 0.54 ms; multi-band factor = 3; Phase-encoding direction: anterior to posterior (AP).

#### Field Map

The field map was acquired to correct the magnetic field distortion using a two-dimensional spin-echo sequence: 72 slices co-planar with the AC/PC; in-plane resolution = 2 × 2 mm; 2 mm slice thickness; field of view = 200 × 200 mm; TR = 720 ms; TE1/TE2 = 4.92/7.38 ms; flip angle = 60°.

#### Structural MRI

Structural T1w images were collected for the anatomical reference using a three-dimensional magnetization-prepared rapid acquisition gradient echo sequence: 208 sagittal slices; 1 mm slice thickness; isotropic voxel size = 1 × 1 × 1 mm; field of view = 256 × 256 mm; TR = 2530 ms; TE = 2.27 ms; TI = 1100 ms; flip angle = 7°.

### Data preprocessing and analysis

#### Data organization

The Digital Imaging and Communications in Medicine (DICOM) images acquired from the Siemens scanner were converted into the Neuroimaging Informatics Technology Initiative (NIfTI) format and then organized into the Brain Imaging Data Structure (BIDS)^34^ using HeuDiConv (https://github.com/nipy/heudiconv)^35^. The facial features were removed from anatomical T1w images using the PyDeface (https://github.com/poldracklab/pydeface)^36^ for data anonymization.

#### MRI preprocessing

The MRI data were preprocessed using fMRIPrep 20.2.1, a robust preprocessing pipeline for structural and functional MRI built by integrating tools from different neuroimaging packages^37^. In brief, individual structural MRI was intensity corrected, skull stripped, and normalized to ICBM152 nonlinear asymmetrical template using ANTs^38^. Brain tissue segmentation and brain surface reconstruction were then performed by combining FAST^39^ and FreeSurfer^40^. Functional MRI data were corrected for motion, slice timing and susceptibility distortions using MCFLIRT^41^, 3dTshift^42^ and SDCflows^43^, respectively and finally co-registered to the T1w using bbregister^44^. For more details on the fMRIPrep pipeline, see Supplementary Information.

All individual fMRI data preprocessed in native volume space were registered onto the standard fsLR space using the Ciftify toolbox for surface-based analysis^45^. In short, the ciftify_recon_all function was used to register and resample individual surfaces to 32k standard fsLR surfaces via surface-based alignment. The ciftify_subject_fmri function was then used to project functional MRI data onto the fsLR surface. All the codes for the data preprocessing and analysis are available at https://github.com/BNUCNL/HAD-fmri.

#### General linear model for estimating BOLD response for action categories

A general linear model (GLM) was constructed to estimate the BOLD responses for each of the action categories from the fMRI data. As the 180 action categories were cycled once every three runs, we modeled the data from each cycle to estimate the BOLD responses to each category and checked the inter-cycle reliability of the responses. That is, functional data from a cycle were concatenated and then modeled vertex by vertex with a GLM. For each vertex, each trial (i.e., category) was modeled separately by convolving its onset timing function with a canonical hemodynamic response function. The second-order polynomial nuisance regressors were also added to the model for each run to account for the drifting effects. To improve the stability of the coefficients estimates for the noised single-trial data, ridge regression was performed to estimate the coefficients of the GLM with a fixed regularization hyperparameter (alpha = 0.1) for all vertices. The vertex-specific responses (i.e., beta values) estimated for each category were used for further analyses. Note that we did not run the grid search for the optimal regularization hyperparameter because it is very time-consuming for the whole-brain vertex-wise ridge regression. However, further post-hoc analyses showed that fine tuning the parameter within the commonly used range (0.01–1) does not change the results much (Supplementary Fig. S2).

## Data Records

**Fig. 2:**
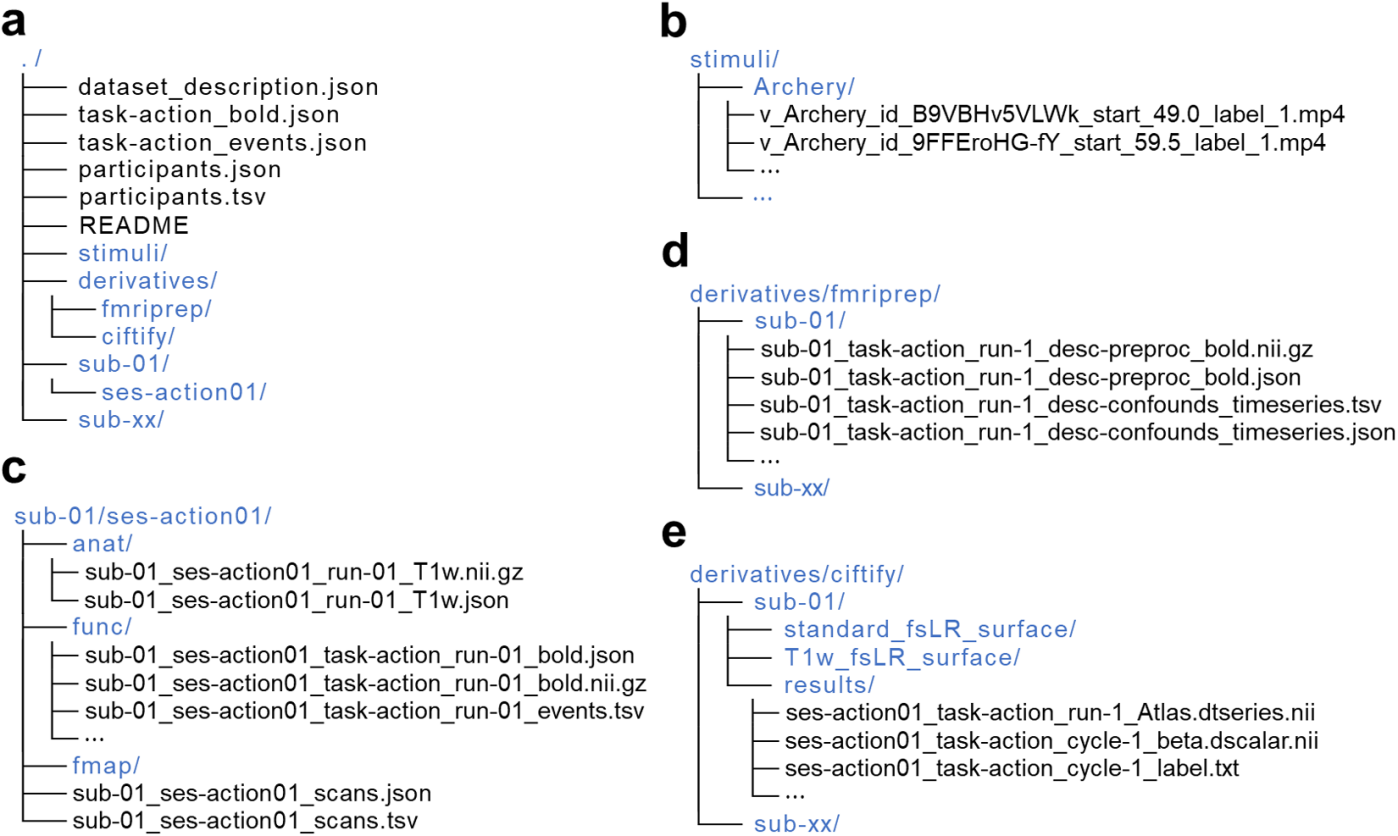
The file structure of Human Action Dataset (HAD). (**a**) The overall directory structure of HAD. (**b**) The file structure of stimulus videos. (**c**) The file structure of the raw data from a sample participant. (**d**) The file structure of the preprocessed data from a sample participant. (**e**) The file structure of the derived surface-based data from a sample participant.

The data were organized according to the Brain-Imaging-Data-Structure (BIDS) Specification version 1.7.0 (Fig. 2a) and can be accessed from the OpenNeuro public repository (https://openneuro.org/datasets/ds004488)^46^. The video clips stimuli were stored in “stimuli” directory (Fig. 2b). The raw data of each subject were stored in “sub-<ID>” directories (Fig. 2c). The preprocessed volume data and the derived surface-based data were stored in “derivatives/fmriprep” and “derivatives/ciftify” directories (Fig. 2d,e), respectively.

As both the raw and the preprocessed data were well organized according to the BIDS which are familiar to most readers, below we only describe the “stimuli” and “derivatives/ciftify” directories in detail.

### Video clips stimuli

The video clips stimuli selected from HACS are deposited in the “stimuli” folder. Each of the 180 action categories holds a folder in which 120 unique video clips are stored (Fig. 2b).

### Preprocessed surface data from ciftify

The preprocessed surface-based data for each functional run are saved as “sub-<subID>/results/ses-action01_task-action_run-<index>_Atlas.dtseries.nii” under the “results” folder (Fig. 2e). The standard and native fsLR surface can be found in the “standard_fsLR_surface” and “T1w_fsLR_surface” folders, respectively. The brain activation data derived from GLM analyses are saved as “sub-<subID>/results/ses-action01_task-action_cycle-<cycleIndex>_beta.dscalar.nii” for each cycle data (Fig. 2e). The auxiliary information about labels or conditions can be found in “ses-action01_task-action_cycle-<cycleIndex>_label.txt”.

## Technical Validation

### Participants show good control in head motion and engage well with the task

**Fig. 3:**
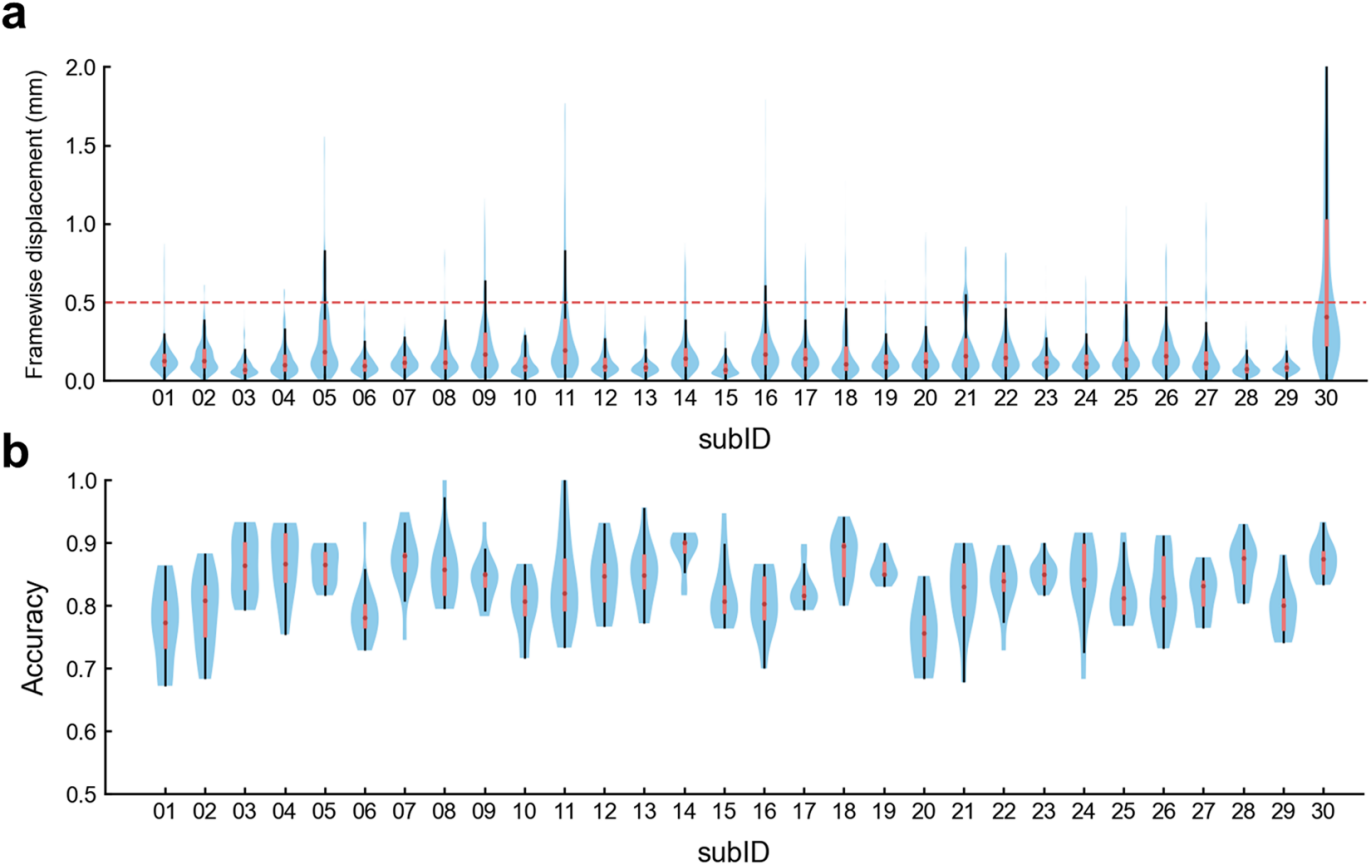
Participants show good control in head motion and engage well with the task. (**a**) The head motion measured by framewise displacement (FD) for individual participants. The violin plots show the distribution of FD for each participant. (**b**) The recognition accuracy in individual participants. The violin plots show the distribution of recognition accuracy from each run for each participant.

The head motion of the participants was quantified with the framewise displacement (FD) metric, which measures instantaneous head motion by comparing the motion between the current and the previous volume^47^. As shown in Fig. 3a, all participants except sub-30 show very few volumes with FD larger than 0.5 mm, which is often used as a criterion to identify the volume with large head motion in the literature^47^. The median of individual FD across all volumes is less than 0.2 mm for all participants except sub-30. The results indicate that participants show good control in head motion when they performed the experiment. What’s more, participants engage well with the task. The average response rate is 94.6% across participants; participants exhibit successful recognition performance: The average recognition accuracy is 83.4% across participants (Fig. 3b).

### The fMRI signal from visual cortex shows high contrast-to-noise ratio for HCAS clips

**Fig. 4:**
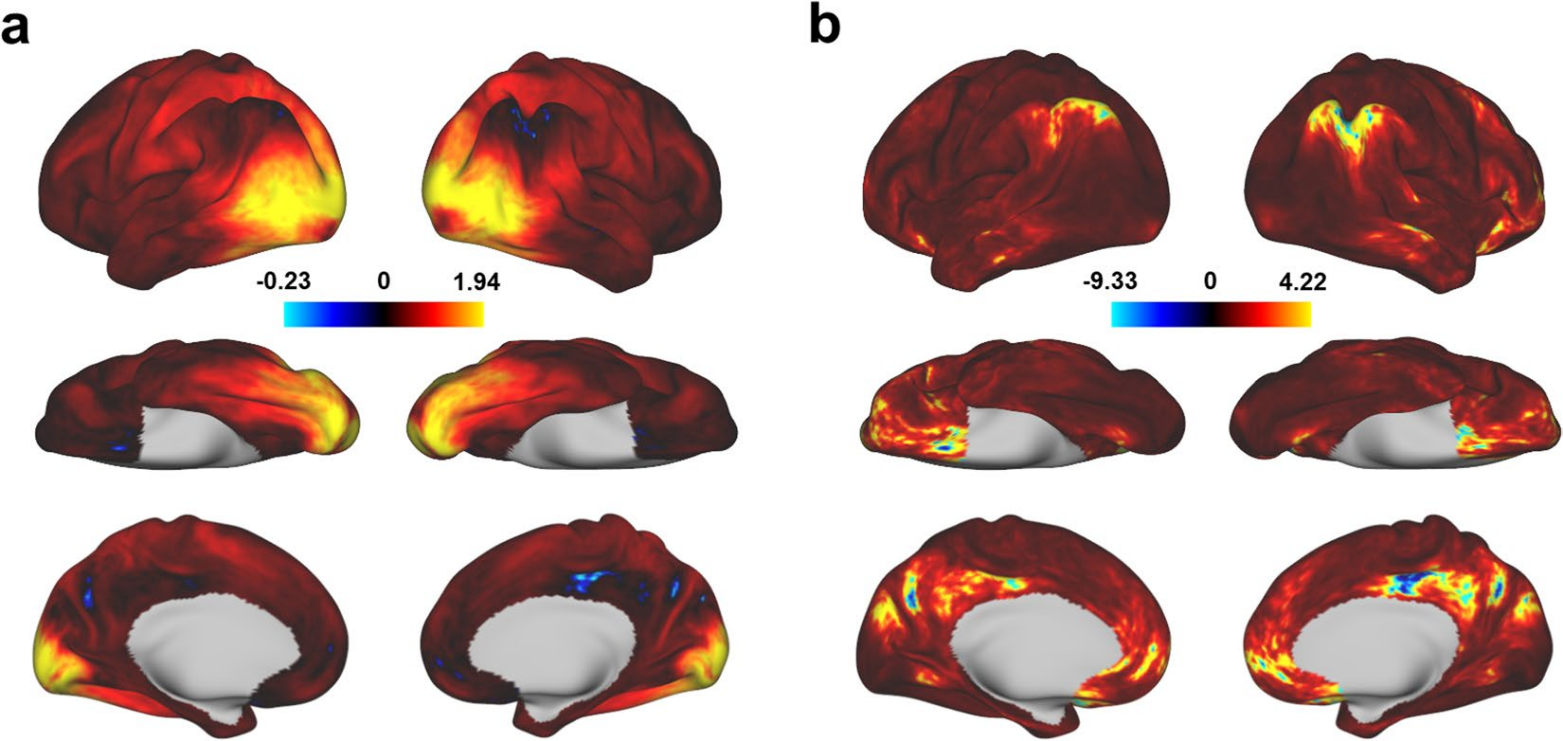
The group contrast-to-noise ratio (CNR) maps in response to HACS clips. The CNR was calculated for each vertex on the standard fsLR surface. (**a**) The group averaged CNR maps across participants. (**b**) The coefficient of variation CNR maps, defined as the ratio of the standard deviation to the mean of the CNR across participants.

We evaluated the contrast-to-noise ratio (CNR) of our fMRI data to check if the HACS clips can induce desired signal changes in each vertex across the cortical surface. The CNR was calculated as the averaged beta values across all categories of stimuli divided by the temporal standard deviation of the residual time series from GLM models. As shown in Fig. 4a, the whole visual cortex, including dorsal, lateral, and ventral pathways, shows high CNR in response to the HACS clips. The mean value of CNR is 0.34 across the whole surface vertices and 0.62 across the visual area vertices defined by the multimodal parcellation atlas^48^, which is a reasonable range for an event-related design^49,50^. Moreover, individual participants show consistent CNR maps (Supplementary Fig. S3). The interindividual variability of the CNR was further characterized by the coefficient of variation (CV). It is revealed that the visual cortex shows a lower CV compared to the non-visual cortex (Fig. 4b). These results indicate that the fMRI signal of visual cortex shows high and consistent CNR in response to HACS clips under our experimental protocols.

### The visual cortex shows reliable responses for the 180 actions categories

**Fig. 5:**
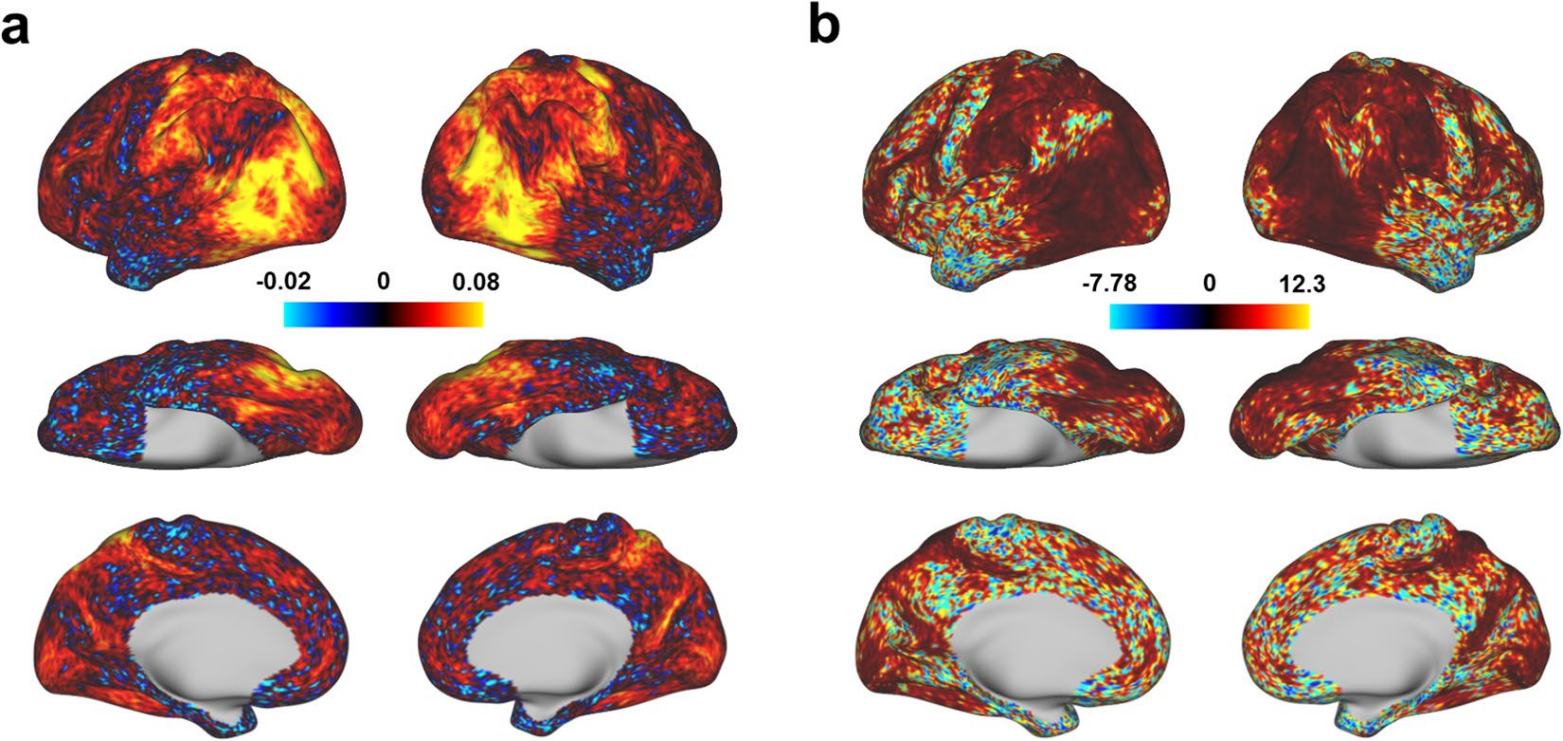
The test-retest reliability maps of BOLD responses for the 180 action categories. The test-retest reliability was computed between the odd and even cycles within each participant. (**a**) The group averaged reliability maps across participants. (**b**) The coefficient of variation reliability maps, defined as the ratio of the standard deviation to the mean across participants.

Next, we assessed the test-retest reliability of BOLD responses for the 180 action categories. As the 180 action categories were repeated four times by cycling every three runs in each session, we computed the Pearson correlation between the brain responses of the 180 categories from the odd and even cycles within each participant to measure the test-retest reliability. As expected, both the lateral stream and the dorsal stream, which are pivotal to action recognition^14–17^, present higher test-retest reliability in response to the 180 categories of actions than other brain areas (Fig. 5a). The reliability maps are consistent across the individual participants (Supplementary Fig. S4). The CV of the individual test-retest reliability maps reveals that the visual cortex shows lower CV values compared to other brain regions (Fig. 5b). Since the participants have reliably performed key pressing in judging if each clip is sport or non-sport, the hand motor areas also show high reliability and low CV. However, the early visual cortex does not show high reliability because no clips are repeatedly presented in different cycles.

### The data can reveal brain areas that show consistent responses to human actions across individuals

**Fig. 6:**
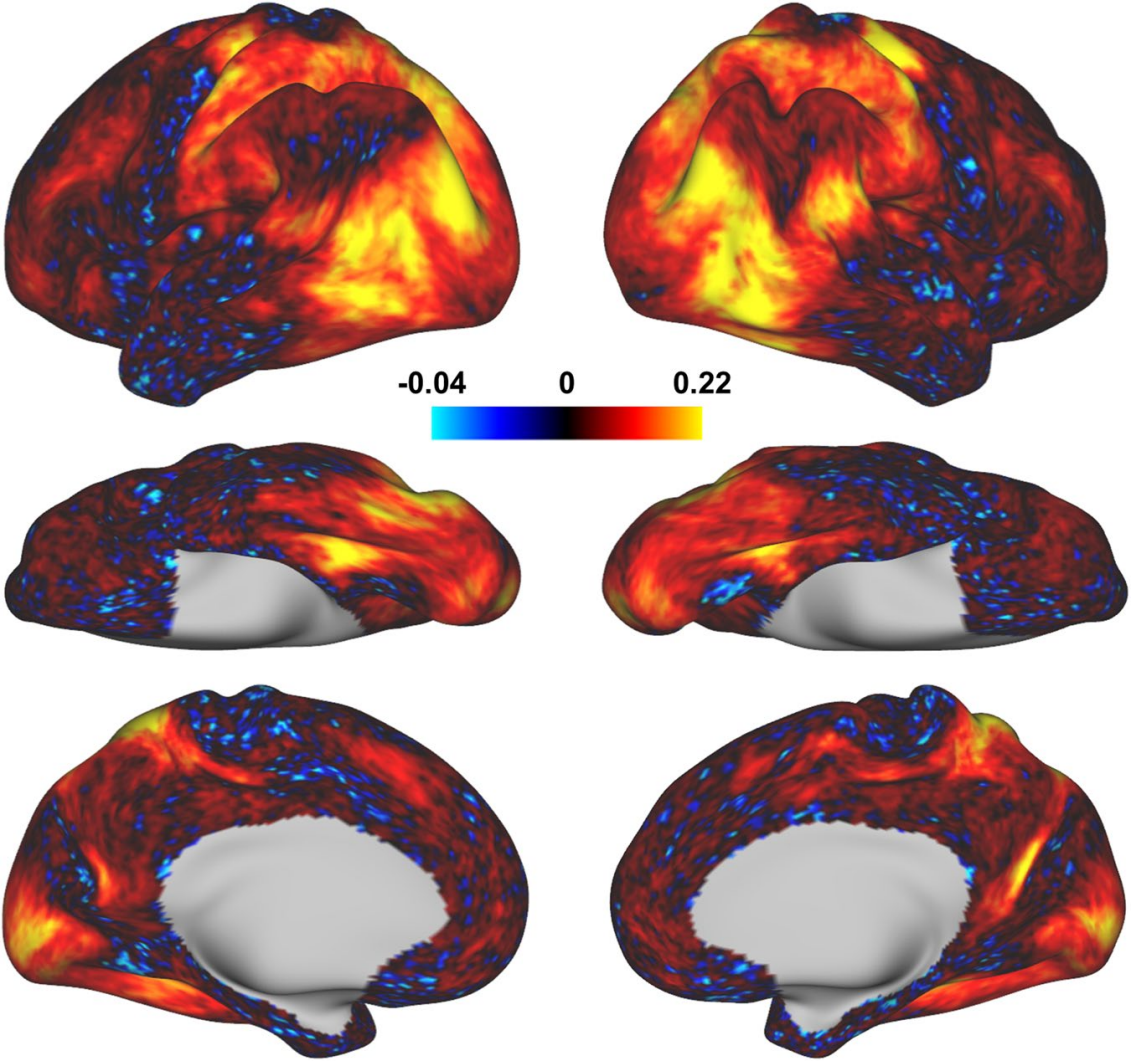
The group inter-subject correlation (ISC) of action category-selective response profiles. The group ISC was produced by averaging the individual ISC, which was computed as Pearson correlation between response profiles per participant and the averaged response profiles from the remaining 29 participants.

An inter-subject correlation (ISC) analysis was performed to validate that our dataset can reveal consistent action category-selective response profiles across participants. ISC has been widely used to localize consistent brain areas across individuals by measuring the consistency of stimulus-locked responses across individuals^51,52^. Here, the ISC was measured for each participant by calculating the Pearson correlation between her/his category-specific response profiles (i.e., beta series) with the averaged category-specific response profiles from the remaining 29 participants. The group ISC was then derived by averaging the individual ISC. As shown in Fig. 6, the spatial patterns of ISC are revealed to be very similar to the test-retest reliability analysis on the individual participant. The early visual cortex, responsible for processing low-level visual features, shows low ISC while the lateral stream and dorsal stream, devoted to processing visual motion and category semantic information^14–17^, show high ISC. Altogether, these results indicate that the recorded neural response profiles to the observed human actions are not only reliable within participants but also consistent across participants.

### The data can characterize the representation similarity for the observed human actions

**Fig. 7:**
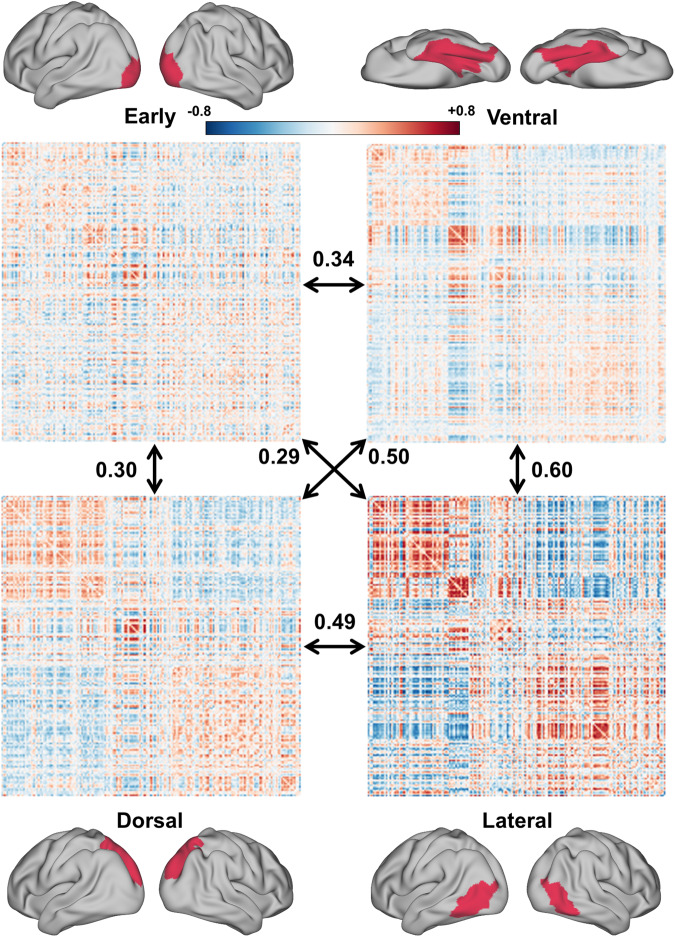
Representational dissimilarity matrices (RDMs) of 180 human action categories computed for the different visual pathways. The RDM was constructed for each participant by computing the Pearson correlation between the multi-voxel activity patterns from 180 categories in the different visual pathways and then averaged across participants. The RDMs from different visual pathways were quantitatively evaluated by the Spearman correlation among them. The axis labels (i.e., action category) of the RDM can be found in Supplementary Table 2.

HAD captures brain responses to observed human actions from a variety of real-world contexts, making it a good resource for investigating the neural representation similarity of the observed human actions. We conducted a representational similarity analysis (RSA)^53^ to validate that multi-voxel activity patterns from the data represent a rich semantic structure of action categories. Specifically, the representational dissimilarity matrix (RDM) of the 180 categories was constructed by computing the Pearson correlation between the multi-voxel activity patterns from each category in different visual pathways. The early, dorsal, lateral, and ventral visual pathways were defined according to the multimodal parcellation atlas^48^. Visual inspection indicates that the RDMs from the four visual pathways show distinct patterns. The RDMs from these pathways were then quantitatively compared by computing the Spearman correlation among them. Two notable findings are revealed here (Fig. 7). First, the RDM from early visual areas is less similar to the RDMs from the three high-level visual pathways as it mainly encodes relatively low-level visual features. Second, the RDM from the lateral pathway shows a larger similarity to that from the ventral pathway instead of the dorsal pathway. These results indicate that the visual pathways show distinct representational similarities for the observed human actions and invite further models of action similarities to elucidate the distinct representation structure of observed human actions in these visual pathways.

## Usage Notes

The diverse and extensive stimulus categories and exemplars in HAD provide unique opportunities for exploring the neural basis of human action recognition. First, the data are well-suited for examining the functional organization of the observed human action in the brain. Particularly, data-driven approaches with large-scale datasets have great potential to discover the representative space of the observed human actions and their organization principles across the cortical surface^5,54–56^. Second, in the future, we and the users can add new annotations to the rich HAD stimuli and make use of this dataset to test more interesting hypotheses on visual action representation^57–60^. Annotating the visual, semantic, and social features of the same stimuli set will help us disentangle the representations of these distinct but correlated feature spaces^4,56^.

While we believe HAD offers unique opportunities to search on the neural basis of human action recognition, we would also like to acknowledge its limitations. First, as previously mentioned, no video clips were repeated in the experiment. This will lead to inaccurate estimates for the BOLD responses of single clips. As a result, the data are not quite fit for exploring the neural representation of a single clip. Second, although a rapid event-related fMRI paradigm was used, sluggish fMRI signals are incapable of resolving neural dynamics for processing dynamic actions. For this, we are conducting a MEG experiment with the same participants and stimuli as HAD. We hope the added MEG measurement will help resolve the spatiotemporal neural dynamics of human action recognition^61,62^.

## Supplementary information

Supplementary Information

Supplementary Figures

Supplementary Table 1

Supplementary Table 2

## Data Availability

All codes for the experimental design, data organization, and technique validation are available at https://github.com/BNUCNL/HAD-fmri. Preprocessing was performed using fMRIPrep version 20.2.1 (https://fmriprep.org). Grayordinate-based (CIFTI format) brain activation analysis was performed by combining the Ciftify (https://github.com/edickie/ciftify) and HCP pipelines (https://github.com/Washington-University/HCPpipelines).
