# Supplementary material for: A large-scale fMRI dataset for human action recognition
Source: Sci Data. 2023 Jun 27;10:415. doi: 10.1038/s41597-023-02325-6 (PMC10300118; doi:10.1038/s41597-023-02325-6)
Supplement: Supplementary file 2 — Supplementary Figures [file 41597_2023_2325_MOESM2_ESM.docx]

**Supplementary Figures**

Supplementary Fig. S1 2

Supplementary Fig. S2 3

Supplementary Fig. S3 4

Supplementary Fig. S4 5





**Supplementary Fig. S1. The number of unrecognized samples for each of the categories visually detected by human raters.** Some action categories (e.g., using the balance beam and rock-paper-scissors) show much more unrecognized samples than others (e.g., knitting and ping-pong), indicating that visual inspection is very necessary to select qualified stimuli for the subsequent fMRI experiment.


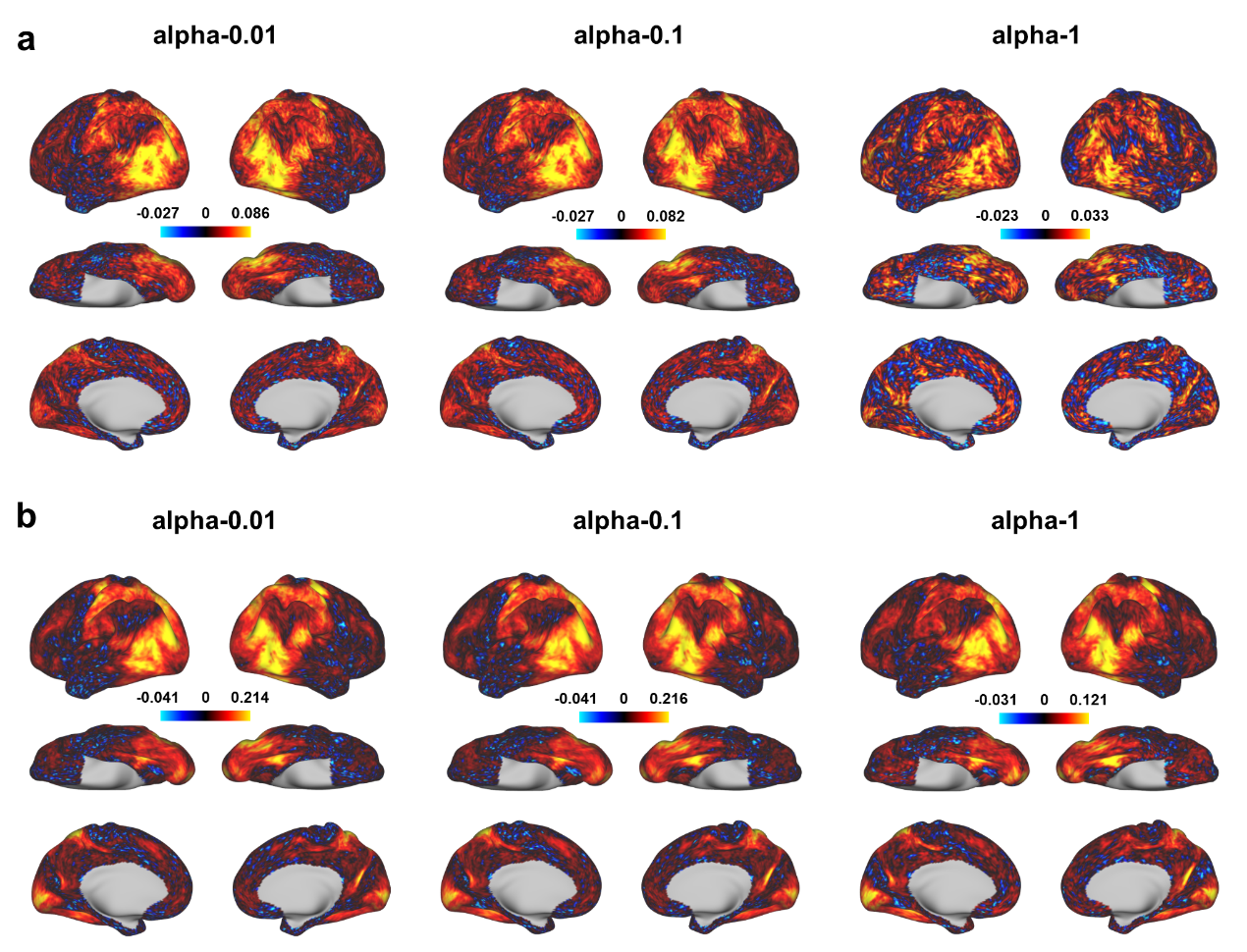


**Supplementary Fig. S2. Changing the regularization parameter(alpha) of ridge regression within the commonly used range (0.01-1) does not change the results. a**, The group average test-retest reliability maps for the BOLD responses estimated with different alpha. **b**, The group average inter-subject correlation maps for the BOLD responses estimated with different alpha.


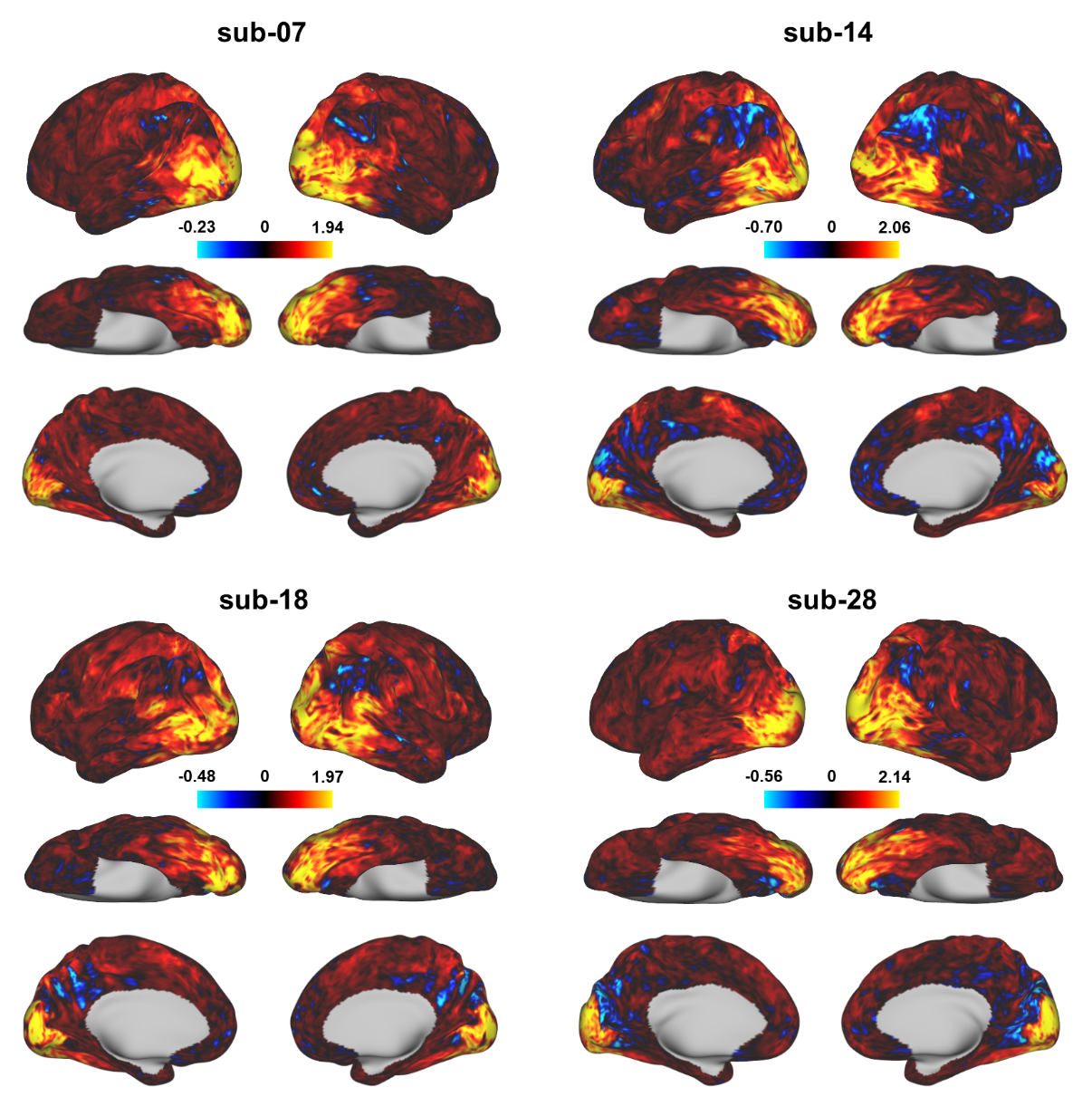


**Supplementary Fig. S3. The individual maps of contrast-to-noise ratio (CNR) from four typical participants.** The CNR maps show consistent patterns across the participants. As expected, the visual cortex shows higher CNR than other parts of the cortex.


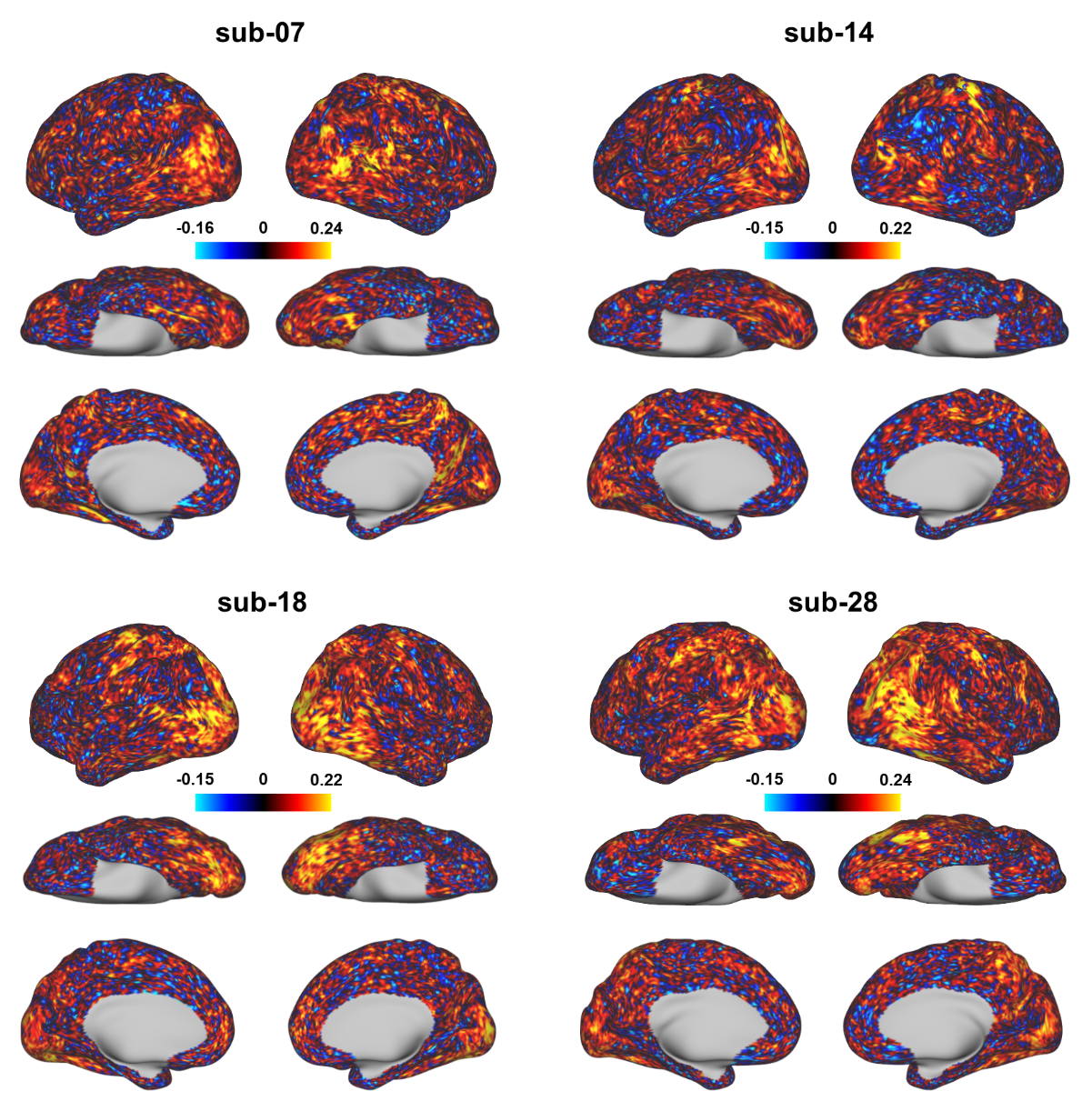


**Supplementary Fig. S4. The individual maps of test-retest reliability from four typical participants.** The reliability maps are highly consistent across participants. And, the ventral, dorsal and lateral visual pathways show higher test-retest reliability compared to the non-visual pathways.
